# Supplementary material for: Design and Simulation of a System-in-Package Chip for Combined Navigation
Source: Micromachines (Basel). 2024 Jan 23;15(2):167. doi: 10.3390/mi15020167 (PMC10892356; doi:10.3390/mi15020167)
Supplement: Supplementary file 1 [file micromachines-15-00167-s001.zip › micromachines-2832037-supplementary.pdf]

Frequency	U3_1V8_G	U3_1V8_GND (Z para - Imaginary)
100000	0.036056	-0.03285
109648	0.036065	-0.02994
120226	0.036071	-0.02729
131826	0.036078	-0.02486
144544	0.036088	-0.02265
158489	0.0361	-0.02063
173780	0.036114	-0.01879
190546	0.03613	-0.01711
208930	0.036151	-0.01557
229087	0.036169	-0.01417
251189	0.036192	-0.01289
275423	0.036219	-0.01173
301995	0.036248	-0.01067
331131	0.036282	-0.0097
363078	0.036318	-0.00882
398107	0.036359	-0.00802
436516	0.036403	-0.00729
478630	0.036451	-0.00664
524807	0.036502	-0.00604
575440	0.036556	-0.00551
630957	0.036614	-0.00503
691831	0.036676	-0.00461
758578	0.03674	-0.00423
831764	0.036808	-0.0039
912011	0.03688	-0.00362
1.00E+06	0.036955	-0.00338
1.10E+06	0.037035	-0.00318
1.20E+06	0.03712	-0.00303
1.32E+06	0.037209	-0.00293
1.45E+06	0.0373	-0.00288
1.58E+06	0.037393	-0.00288
1.74E+06	0.037482	-0.00296
1.91E+06	0.037559	-0.00311
2.09E+06	0.037612	-0.00334
2.29E+06	0.037619	-0.00368
2.51E+06	0.037552	-0.00414
2.75E+06	0.037364	-0.00472
3.02E+06	0.036998	-0.00541
3.31E+06	0.036378	-0.00619
3.63E+06	0.035427	-0.007
3.98E+06	0.034081	-0.00772
4.37E+06	0.032325	-0.00818
4.79E+06	0.030224	-0.00822
5.25E+06	0.027927	-0.0077
5.75E+06	0.025636	-0.00656
6.31E+06	0.023541	-0.00486

6.92E+06	0.02177	-0.0027
7.59E+06	0.020377	-0.00021
8.32E+06	0.019378	0.002538
9.12E+06	0.018794	0.005475
1.00E+07	0.01866	0.008536
1.04E+07	0.018751	0.009858
1.08E+07	0.018929	0.011132
1.12E+07	0.01919	0.012352
1.16E+07	0.019533	0.013514
1.20E+07	0.019959	0.014609
1.24E+07	0.020469	0.015623
1.28E+07	0.021061	0.016537
1.32E+07	0.021728	0.017327
1.36E+07	0.022453	0.017963
1.40E+07	0.023207	0.018415
1.44E+07	0.02394	0.018658
1.48E+07	0.024586	0.018683
1.52E+07	0.02507	0.018513
1.56E+07	0.025324	0.018204
1.60E+07	0.025308	0.017845
1.64E+07	0.025028	0.017531
1.68E+07	0.024528	0.017346
1.72E+07	0.02388	0.017339
1.76E+07	0.023157	0.017523
1.80E+07	0.022421	0.017886
1.84E+07	0.021716	0.018397
1.88E+07	0.021069	0.019023
1.92E+07	0.020489	0.019733
1.96E+07	0.019979	0.020498
2.00E+07	0.019534	0.021298
2.04E+07	0.019147	0.022119
2.08E+07	0.018806	0.022955
2.12E+07	0.018509	0.023784
2.16E+07	0.018246	0.02462
2.20E+07	0.018012	0.025458
2.24E+07	0.017807	0.026295
2.28E+07	0.017627	0.02713
2.32E+07	0.017471	0.02796
2.36E+07	0.017334	0.028785
2.40E+07	0.017216	0.029604
2.44E+07	0.017115	0.030415
2.48E+07	0.017028	0.031219
2.52E+07	0.016954	0.032016
2.56E+07	0.016891	0.032804
2.60E+07	0.016838	0.033585
2.64E+07	0.016794	0.034358
2.68E+07	0.016757	0.035123

2.72E+07	0.016727	0.035881
2.76E+07	0.016702	0.036633
2.80E+07	0.016683	0.037378
2.84E+07	0.016667	0.038117
2.88E+07	0.016656	0.03885
2.92E+07	0.016647	0.039578
2.96E+07	0.016642	0.0403
3.00E+07	0.016639	0.041018
3.04E+07	0.016639	0.041731
3.08E+07	0.01664	0.04244
3.12E+07	0.016644	0.043146
3.16E+07	0.016649	0.043847
3.20E+07	0.016656	0.044544
3.24E+07	0.016664	0.045239
3.28E+07	0.016673	0.04593
3.32E+07	0.016683	0.046617
3.36E+07	0.016695	0.047302
3.40E+07	0.016707	0.047984
3.44E+07	0.01672	0.048663
3.48E+07	0.016734	0.04934
3.52E+07	0.016748	0.050014
3.56E+07	0.016763	0.050685
3.60E+07	0.016779	0.051355
3.64E+07	0.016795	0.052022
3.68E+07	0.016812	0.052687
3.72E+07	0.016829	0.053351
3.76E+07	0.016846	0.054012
3.80E+07	0.016864	0.054671
3.84E+07	0.016884	0.055341
3.88E+07	0.016901	0.056008
3.92E+07	0.01692	0.056663
3.96E+07	0.016939	0.057314
4.00E+07	0.016958	0.057965
4.04E+07	0.016978	0.058614
4.08E+07	0.016997	0.059261
4.12E+07	0.017017	0.059908
4.16E+07	0.017037	0.060553
4.20E+07	0.017057	0.061196
4.24E+07	0.017078	0.061839
4.28E+07	0.017098	0.06248
4.32E+07	0.017119	0.063121
4.36E+07	0.017139	0.06376
4.40E+07	0.01716	0.064399
4.44E+07	0.017181	0.065036
4.48E+07	0.017202	0.065672
4.52E+07	0.017223	0.066308
4.56E+07	0.017244	0.066942

4.60E+07	0.017265	0.067576
4.64E+07	0.017286	0.068209
4.68E+07	0.017307	0.068841
4.72E+07	0.017328	0.069472
4.76E+07	0.017349	0.070102
4.80E+07	0.01737	0.070732
4.84E+07	0.017392	0.071361
4.88E+07	0.017413	0.071989
4.92E+07	0.017434	0.072616
4.96E+07	0.017455	0.073243
5.00E+07	0.017477	0.073869
5.20E+07	0.017582	0.07699
5.40E+07	0.017688	0.080097
5.60E+07	0.017794	0.083192
5.80E+07	0.017899	0.086275
6.00E+07	0.018004	0.089349
6.20E+07	0.018109	0.092414
6.40E+07	0.018212	0.095471
6.60E+07	0.018315	0.09852
6.80E+07	0.018417	0.101562
7.00E+07	0.018518	0.104598
7.20E+07	0.018618	0.107628
7.40E+07	0.018716	0.110653
7.60E+07	0.018814	0.113674
7.80E+07	0.018911	0.116689
8.00E+07	0.019007	0.119701
8.20E+07	0.019102	0.122709
8.40E+07	0.019196	0.125713
8.60E+07	0.019289	0.128714
8.80E+07	0.019382	0.131712
9.00E+07	0.019474	0.134707
9.20E+07	0.019564	0.1377
9.40E+07	0.019655	0.14069
9.60E+07	0.019744	0.143678
9.80E+07	0.019833	0.146663
1.00E+08	0.019921	0.149647
1.02E+08	0.020009	0.152629
1.04E+08	0.020096	0.155608
1.06E+08	0.020182	0.158587
1.08E+08	0.020268	0.161563
1.10E+08	0.020353	0.164538
1.12E+08	0.020438	0.167512
1.14E+08	0.020523	0.170484
1.16E+08	0.020607	0.173455
1.18E+08	0.02069	0.176425
1.20E+08	0.020774	0.179394
1.22E+08	0.020856	0.182361

1.24E+08	0.020939	0.185328
1.26E+08	0.021021	0.188293
1.28E+08	0.021103	0.191258
1.30E+08	0.021184	0.194222
1.32E+08	0.021266	0.197185
1.34E+08	0.021346	0.200147
1.36E+08	0.021427	0.203108
1.38E+08	0.021507	0.206069
1.40E+08	0.021587	0.209029
1.42E+08	0.021667	0.211988
1.44E+08	0.021747	0.214947
1.46E+08	0.021826	0.217905
1.48E+08	0.021905	0.220863
1.50E+08	0.021984	0.22382
1.52E+08	0.022063	0.226776
1.54E+08	0.022141	0.229733
1.56E+08	0.02222	0.232688
1.58E+08	0.022298	0.235644
1.60E+08	0.022376	0.238599
1.62E+08	0.022453	0.241553
1.64E+08	0.022531	0.244508
1.66E+08	0.022608	0.247461
1.68E+08	0.022686	0.250415
1.70E+08	0.022763	0.253368
1.72E+08	0.02284	0.256322
1.74E+08	0.022917	0.259274
1.76E+08	0.022993	0.262227
1.78E+08	0.02307	0.26518
1.80E+08	0.023146	0.268132
1.82E+08	0.023223	0.271084
1.84E+08	0.023299	0.274036
1.86E+08	0.023375	0.276988
1.88E+08	0.023451	0.27994
1.90E+08	0.023526	0.282891
1.92E+08	0.023602	0.285843
1.94E+08	0.023678	0.288795
1.96E+08	0.023753	0.291746
1.98E+08	0.023829	0.294698
2.00E+08	0.023904	0.297649
2.02E+08	0.023979	0.3006
2.04E+08	0.024054	0.303552
2.06E+08	0.02413	0.306503
2.08E+08	0.024205	0.309455
2.10E+08	0.024279	0.312407
2.12E+08	0.024354	0.315358
2.14E+08	0.024429	0.31831
2.16E+08	0.024504	0.321262

2.18E+08	0.024578	0.324214
2.20E+08	0.024653	0.327166
2.22E+08	0.024727	0.330118
2.24E+08	0.024802	0.33307
2.26E+08	0.024876	0.336023
2.28E+08	0.02495	0.338975
2.30E+08	0.025025	0.341928
2.32E+08	0.025099	0.344881
2.34E+08	0.025173	0.347834
2.36E+08	0.025247	0.350788
2.38E+08	0.025321	0.353741
2.40E+08	0.025395	0.356695
2.42E+08	0.025469	0.359649
2.44E+08	0.025543	0.362604
2.46E+08	0.025617	0.365558
2.48E+08	0.025691	0.368513
2.50E+08	0.025764	0.371468
2.52E+08	0.025838	0.374424
2.54E+08	0.025912	0.377379
2.56E+08	0.025985	0.380335
2.58E+08	0.026059	0.383292
2.60E+08	0.026133	0.386249
2.62E+08	0.026206	0.389206
2.64E+08	0.02628	0.392163
2.66E+08	0.026353	0.395121
2.68E+08	0.026427	0.398079
2.70E+08	0.0265	0.401037
2.72E+08	0.026574	0.403996
2.74E+08	0.026647	0.406956
2.76E+08	0.02672	0.409915
2.78E+08	0.026794	0.412876
2.80E+08	0.026867	0.415836
2.82E+08	0.02694	0.418797
2.84E+08	0.027013	0.421759
2.86E+08	0.027087	0.42472
2.88E+08	0.02716	0.427683
2.90E+08	0.027233	0.430646
2.92E+08	0.027306	0.433609
2.94E+08	0.027379	0.436573
2.96E+08	0.027452	0.439537
2.98E+08	0.027526	0.442502
3.00E+08	0.027599	0.445467
3.02E+08	0.027672	0.448433
3.04E+08	0.027745	0.4514
3.06E+08	0.027818	0.454367
3.08E+08	0.027891	0.457334
3.10E+08	0.027964	0.460302

3.12E+08	0.028037	0.463271
3.14E+08	0.02811	0.46624
3.16E+08	0.028183	0.46921
3.18E+08	0.028256	0.472181
3.20E+08	0.028329	0.475152
3.22E+08	0.028401	0.478124
3.24E+08	0.028474	0.481096
3.26E+08	0.028547	0.484069
3.28E+08	0.02862	0.487043
3.30E+08	0.028693	0.490017
3.32E+08	0.028766	0.492992
3.34E+08	0.028839	0.495968
3.36E+08	0.028911	0.498944
3.38E+08	0.028984	0.501921
3.40E+08	0.029057	0.504899
3.42E+08	0.02913	0.507877
3.44E+08	0.029202	0.510857
3.46E+08	0.029275	0.513837
3.48E+08	0.029348	0.516817
3.50E+08	0.02942	0.519799
3.52E+08	0.029493	0.522781
3.54E+08	0.029566	0.525764
3.56E+08	0.029638	0.528748
3.58E+08	0.029711	0.531732
3.60E+08	0.029784	0.534718
3.62E+08	0.029856	0.537704
3.64E+08	0.029929	0.540691
3.66E+08	0.030001	0.543679
3.68E+08	0.030074	0.546667
3.70E+08	0.030146	0.549657
3.72E+08	0.030219	0.552647
3.74E+08	0.030291	0.555639
3.76E+08	0.030364	0.558631
3.78E+08	0.030436	0.561624
3.80E+08	0.030509	0.564618
3.82E+08	0.030581	0.567613
3.84E+08	0.030654	0.570608
3.86E+08	0.030726	0.573605
3.88E+08	0.030798	0.576603
3.90E+08	0.030871	0.579601
3.92E+08	0.030943	0.582601
3.94E+08	0.031015	0.585601
3.96E+08	0.031088	0.588603
3.98E+08	0.03116	0.591605
4.00E+08	0.031232	0.594609
4.02E+08	0.031305	0.597613
4.04E+08	0.031377	0.600619

4.06E+08	0.031449	0.603626
4.08E+08	0.031521	0.606633
4.10E+08	0.031593	0.609642
4.12E+08	0.031665	0.612651
4.14E+08	0.031737	0.615662
4.16E+08	0.031809	0.618674
4.18E+08	0.031881	0.621687
4.20E+08	0.031953	0.624701
4.22E+08	0.032025	0.627716
4.24E+08	0.032097	0.630733
4.26E+08	0.032169	0.63375
4.28E+08	0.032241	0.636768
4.30E+08	0.032313	0.639788
4.32E+08	0.032385	0.642809
4.34E+08	0.032457	0.645831
4.36E+08	0.032528	0.648854
4.38E+08	0.0326	0.651879
4.40E+08	0.032672	0.654904
4.42E+08	0.032743	0.657931
4.44E+08	0.032815	0.660959
4.46E+08	0.032886	0.663988
4.48E+08	0.032958	0.667019
4.50E+08	0.033029	0.670051
4.52E+08	0.033101	0.673084
4.54E+08	0.033172	0.676118
4.56E+08	0.033243	0.679153
4.58E+08	0.033315	0.68219
4.60E+08	0.033386	0.685229
4.62E+08	0.033457	0.688268
4.64E+08	0.033528	0.691309
4.66E+08	0.033599	0.694351
4.68E+08	0.03367	0.697394
4.70E+08	0.033741	0.700439
4.72E+08	0.033812	0.703485
4.74E+08	0.033883	0.706533
4.76E+08	0.033954	0.709582
4.78E+08	0.034025	0.712632
4.80E+08	0.034095	0.715684
4.82E+08	0.034166	0.718737
4.84E+08	0.034237	0.721792
4.86E+08	0.034307	0.724848
4.88E+08	0.034378	0.727905
4.90E+08	0.034448	0.730964
4.92E+08	0.034519	0.734024
4.94E+08	0.034589	0.737086
4.96E+08	0.034659	0.74015
4.98E+08	0.03473	0.743215

5.00E+08	0.0348	0.746281
5.05E+08	0.034976	0.753954
5.10E+08	0.035152	0.761636
5.15E+08	0.035328	0.769329
5.20E+08	0.035504	0.777031
5.25E+08	0.035681	0.784744
5.30E+08	0.035859	0.792468
5.35E+08	0.036038	0.800202
5.40E+08	0.036218	0.807947
5.45E+08	0.0364	0.815704
5.50E+08	0.036584	0.823472
5.55E+08	0.036768	0.831251
5.60E+08	0.036953	0.839043
5.65E+08	0.037138	0.846847
5.70E+08	0.037318	0.854662
5.75E+08	0.037493	0.862488
5.80E+08	0.037657	0.870325
5.85E+08	0.037812	0.878175
5.90E+08	0.037971	0.88604
5.95E+08	0.038167	0.893919
6.00E+08	0.038442	0.901832
6.05E+08	0.038522	0.909716
6.10E+08	0.0387	0.917635
6.15E+08	0.038732	0.926028
6.20E+08	0.03891	0.933974
6.25E+08	0.039088	0.941936
6.30E+08	0.039267	0.949912
6.35E+08	0.039446	0.957904
6.40E+08	0.039625	0.965912
6.45E+08	0.039805	0.973936
6.50E+08	0.039986	0.981975
6.55E+08	0.040166	0.990031
6.60E+08	0.040348	0.998104
6.65E+08	0.04053	1.00619
6.70E+08	0.040713	1.0143
6.75E+08	0.040896	1.02243
6.80E+08	0.04108	1.03057
6.85E+08	0.041264	1.03873
6.90E+08	0.04145	1.04691
6.95E+08	0.041636	1.05511
7.00E+08	0.041822	1.06332
7.05E+08	0.04201	1.07156
7.10E+08	0.042198	1.07981
7.15E+08	0.042387	1.08809
7.20E+08	0.042577	1.09638
7.25E+08	0.042768	1.1047
7.30E+08	0.04296	1.11304

7.35E+08	0.043152	1.12139
7.40E+08	0.043346	1.12977
7.45E+08	0.04354	1.13818
7.50E+08	0.043736	1.1466
7.55E+08	0.043933	1.15505
7.60E+08	0.044131	1.16351
7.65E+08	0.044329	1.17201
7.70E+08	0.04453	1.18052
7.75E+08	0.044731	1.18906
7.80E+08	0.044933	1.19763
7.85E+08	0.045137	1.20622
7.90E+08	0.045342	1.21483
7.95E+08	0.045549	1.22347
8.00E+08	0.045756	1.23214
8.05E+08	0.045966	1.24083
8.10E+08	0.046176	1.24955
8.15E+08	0.046388	1.2583
8.20E+08	0.046602	1.26708
8.25E+08	0.046818	1.27588
8.30E+08	0.047034	1.28472
8.35E+08	0.047253	1.29358
8.40E+08	0.047474	1.30247
8.45E+08	0.047696	1.31139
8.50E+08	0.04792	1.32035
8.55E+08	0.048146	1.32933
8.60E+08	0.048373	1.33835
8.65E+08	0.048603	1.3474
8.70E+08	0.048835	1.35648
8.75E+08	0.049069	1.3656
8.80E+08	0.049305	1.37475
8.85E+08	0.049543	1.38393
8.90E+08	0.049784	1.39315
8.95E+08	0.050027	1.40241
9.00E+08	0.050272	1.4117
9.05E+08	0.05052	1.42103
9.10E+08	0.05077	1.4304
9.15E+08	0.051023	1.43981
9.20E+08	0.051279	1.44925
9.25E+08	0.051537	1.45874
9.30E+08	0.051798	1.46827
9.35E+08	0.052063	1.47784
9.40E+08	0.05233	1.48745
9.45E+08	0.0526	1.4971
9.50E+08	0.052873	1.5068
9.55E+08	0.053149	1.51654
9.60E+08	0.053429	1.52633
9.65E+08	0.053713	1.53617

9.70E+08	0.053999	1.54605
9.75E+08	0.05429	1.55598
9.80E+08	0.054584	1.56596
9.85E+08	0.054882	1.57599
9.90E+08	0.055184	1.58607
9.95E+08	0.05549	1.5962
1.00E+09	0.055799	1.60638
1.01E+09	0.056114	1.61662
1.01E+09	0.056432	1.62691
1.02E+09	0.056755	1.63725
1.02E+09	0.057083	1.64765
1.03E+09	0.057415	1.65811
1.03E+09	0.057752	1.66863
1.04E+09	0.058094	1.67921
1.04E+09	0.058441	1.68985
1.05E+09	0.058794	1.70056
1.05E+09	0.059152	1.71132
1.06E+09	0.059516	1.72216
1.06E+09	0.059885	1.73306
1.07E+09	0.060261	1.74403
1.07E+09	0.060643	1.75507
1.08E+09	0.061032	1.76618
1.08E+09	0.061427	1.77736
1.09E+09	0.061828	1.78862
1.09E+09	0.062237	1.79995
1.10E+09	0.062653	1.81136
1.10E+09	0.063077	1.82285
1.11E+09	0.063508	1.83443
1.11E+09	0.063948	1.84608
1.12E+09	0.064395	1.85782
1.12E+09	0.064851	1.86965
1.13E+09	0.065316	1.88156
1.13E+09	0.065789	1.89357
1.14E+09	0.066273	1.90567
1.14E+09	0.066765	1.91786
1.15E+09	0.067268	1.93015
1.15E+09	0.067781	1.94254
1.16E+09	0.068304	1.95503
1.16E+09	0.068839	1.96763
1.17E+09	0.069385	1.98033
1.17E+09	0.069943	1.99315
1.18E+09	0.070513	2.00607
1.18E+09	0.071095	2.01911
1.19E+09	0.071691	2.03227
1.19E+09	0.0723	2.04555
1.20E+09	0.072923	2.05895
1.20E+09	0.07356	2.07248

1.21E+09	0.074213	2.08614
1.21E+09	0.074881	2.09993
1.22E+09	0.075565	2.11386
1.22E+09	0.076266	2.12793
1.23E+09	0.076985	2.14215
1.23E+09	0.077721	2.15651
1.24E+09	0.078476	2.17103
1.24E+09	0.079251	2.1857
1.25E+09	0.080046	2.20053
1.25E+09	0.080862	2.21553
1.26E+09	0.0817	2.2307
1.26E+09	0.08256	2.24605
1.27E+09	0.083445	2.26157
1.27E+09	0.084354	2.27728
1.28E+09	0.085288	2.29319
1.28E+09	0.08625	2.30928
1.29E+09	0.087239	2.32558
1.29E+09	0.088258	2.34209
1.30E+09	0.089307	2.35882
1.30E+09	0.090387	2.37576
1.31E+09	0.091501	2.39293
1.31E+09	0.09265	2.41034
1.32E+09	0.093835	2.428
1.32E+09	0.095058	2.4459
1.33E+09	0.096321	2.46407
1.33E+09	0.097625	2.4825
1.34E+09	0.098974	2.50121
1.34E+09	0.100369	2.5202
1.35E+09	0.101812	2.5395
1.35E+09	0.103306	2.5591
1.36E+09	0.104855	2.57902
1.36E+09	0.10646	2.59927
1.37E+09	0.108126	2.61986
1.37E+09	0.109855	2.64081
1.38E+09	0.111653	2.66212
1.38E+09	0.113523	2.68382
1.39E+09	0.115471	2.70592
1.39E+09	0.117503	2.72843
1.40E+09	0.119625	2.75137
1.40E+09	0.121845	2.77476
1.41E+09	0.124172	2.79862
1.41E+09	0.126619	2.82297
1.42E+09	0.129199	2.84784
1.42E+09	0.131931	2.87324
1.43E+09	0.134838	2.89919
1.43E+09	0.137951	2.92573
1.44E+09	0.141313	2.95286

1.44E+09	0.144982	2.98059
1.45E+09	0.149031	3.00891
1.45E+09	0.153551	3.03774
1.46E+09	0.158619	3.06694
1.46E+09	0.16421	3.0962
1.47E+09	0.17002	3.12518
1.47E+09	0.175323	3.15376
1.48E+09	0.179339	3.18258
1.48E+09	0.182055	3.21277
1.49E+09	0.184256	3.24501
1.49E+09	0.18672	3.27922
1.50E+09	0.189802	3.31506
1.50E+09	0.193559	3.35225
1.51E+09	0.197939	3.39065
1.51E+09	0.202878	3.43021
1.52E+09	0.208327	3.47094
1.52E+09	0.214258	3.51291
1.53E+09	0.220661	3.55616
1.53E+09	0.227539	3.60079
1.54E+09	0.234906	3.64686
1.54E+09	0.242785	3.69447
1.55E+09	0.251204	3.74372
1.55E+09	0.260201	3.79472
1.56E+09	0.269818	3.84757
1.56E+09	0.280105	3.90239
1.57E+09	0.291119	3.95933
1.57E+09	0.302924	4.01851
1.58E+09	0.315591	4.0801
1.58E+09	0.329204	4.14426
1.59E+09	0.343854	4.21117
1.59E+09	0.359646	4.28103
1.60E+09	0.376698	4.35406
1.60E+09	0.395144	4.43051
1.61E+09	0.415137	4.51062
1.61E+09	0.43685	4.5947
1.62E+09	0.460484	4.68307
1.62E+09	0.486267	4.77606
1.63E+09	0.514463	4.87409
1.63E+09	0.545375	4.97758
1.64E+09	0.579359	5.08702
1.64E+09	0.616827	5.20295
1.65E+09	0.658263	5.32597
1.65E+09	0.704234	5.45678
1.66E+09	0.755414	5.59612
1.66E+09	0.812603	5.74486
1.67E+09	0.876757	5.90396
1.67E+09	0.949026	6.07452

1.68E+09	1.0308	6.25778
1.68E+09	1.12378	6.45512
1.69E+09	1.23004	6.66812
1.69E+09	1.35215	6.89856
1.70E+09	1.49331	7.14841
1.70E+09	1.65753	7.41989
1.71E+09	1.84991	7.7154
1.71E+09	2.07693	8.03752
1.72E+09	2.34695	8.38888
1.72E+09	2.67082	8.77191
1.73E+09	3.06272	9.18844
1.73E+09	3.5413	9.6389
1.74E+09	4.13119	10.1208
1.74E+09	4.86475	10.6262
1.75E+09	5.78396	11.137
1.75E+09	6.94149	11.6171
1.76E+09	8.39842	11.9994
1.76E+09	10.213	12.1663
1.77E+09	12.4092	11.9283
1.77E+09	14.9099	11.0168
1.78E+09	17.4372	9.14349
1.78E+09	19.4557	6.18336
1.79E+09	20.3285	2.42694
1.79E+09	19.7256	-1.39653
1.80E+09	17.8981	-4.52137
1.80E+09	15.461	-6.58587
1.81E+09	12.9751	-7.66509
1.81E+09	10.7531	-8.03145
1.82E+09	8.89663	-7.9565
1.82E+09	7.39488	-7.63881
1.83E+09	6.19524	-7.20401
1.83E+09	5.23854	-6.72536
1.84E+09	4.47236	-6.24318
1.84E+09	3.8543	-5.77834
1.85E+09	3.35141	-5.34068
1.85E+09	2.93848	-4.9339
1.86E+09	2.59632	-4.55844
1.86E+09	2.3103	-4.21305
1.87E+09	2.06921	-3.89569
1.87E+09	1.86438	-3.60401
1.88E+09	1.68907	-3.33566
1.88E+09	1.538	-3.08838
1.89E+09	1.40698	-2.86008
1.89E+09	1.29268	-2.64885
1.90E+09	1.19241	-2.45299
1.90E+09	1.104	-2.27098
1.91E+09	1.02567	-2.10146

1.91E+09	0.955958	-1.94323
1.92E+09	0.893662	-1.79523
1.92E+09	0.837775	-1.65649
1.93E+09	0.787454	-1.5262
1.93E+09	0.74199	-1.40358
1.94E+09	0.700782	-1.28798
1.94E+09	0.663319	-1.1788
1.95E+09	0.629164	-1.07551
1.95E+09	0.597943	-0.97762
1.96E+09	0.569331	-0.8847
1.96E+09	0.543049	-0.79638
1.97E+09	0.518853	-0.71229
1.97E+09	0.496531	-0.63212
1.98E+09	0.475898	-0.55558
1.98E+09	0.45679	-0.48242
1.99E+09	0.439065	-0.4124
1.99E+09	0.422594	-0.34529
2.00E+09	0.407268	-0.28091
2.00E+09	0.392986	-0.21906
2.01E+09	0.367213	-0.10234
2.02E+09	0.344683	0.006054
2.03E+09	0.324929	0.107123
2.04E+09	0.307586	0.201733
2.05E+09	0.292375	0.290636
2.06E+09	0.279103	0.374491
2.07E+09	0.26766	0.453879
2.08E+09	0.258042	0.529306
2.09E+09	0.25039	0.601191
2.10E+09	0.245053	0.6698
2.11E+09	0.242681	0.73504
2.12E+09	0.244173	0.79594
2.13E+09	0.249781	0.849715
2.14E+09	0.255926	0.89209
2.15E+09	0.25327	0.924168
2.16E+09	0.238281	0.957395
2.17E+09	0.219477	0.998572
2.18E+09	0.203512	1.04469
2.19E+09	0.191352	1.09183
2.20E+09	0.182086	1.13813
2.21E+09	0.174789	1.18296
2.22E+09	0.168827	1.22621
2.23E+09	0.163799	1.26793
2.24E+09	0.159455	1.30824
2.25E+09	0.15563	1.34725
2.26E+09	0.152214	1.38508
2.27E+09	0.149131	1.42181
2.28E+09	0.146327	1.45754

2.29E+09	0.143761	1.49234
2.30E+09	0.141403	1.52628
2.31E+09	0.139227	1.55942
2.32E+09	0.137215	1.59182
2.33E+09	0.13535	1.62353
2.34E+09	0.133621	1.65459
2.35E+09	0.132017	1.68505
2.36E+09	0.13053	1.71494
2.37E+09	0.129153	1.7443
2.38E+09	0.12788	1.77316
2.39E+09	0.12671	1.80157
2.40E+09	0.125639	1.82954
2.41E+09	0.124669	1.85711
2.42E+09	0.123802	1.88431
2.43E+09	0.123047	1.91115
2.44E+09	0.122413	1.93768
2.45E+09	0.121919	1.96391
2.46E+09	0.121595	1.98987
2.47E+09	0.121482	2.01558
2.48E+09	0.121645	2.04103
2.49E+09	0.122178	2.0662
2.50E+09	0.123206	2.09099
2.51E+09	0.124861	2.11518
2.52E+09	0.127157	2.13833
2.53E+09	0.129689	2.15979
2.54E+09	0.131327	2.17931
2.55E+09	0.130812	2.19782
2.56E+09	0.128218	2.21705
2.57E+09	0.124837	2.23784
2.58E+09	0.121725	2.25983
2.59E+09	0.119232	2.28242
2.60E+09	0.117328	2.30519
2.61E+09	0.115885	2.32792
2.62E+09	0.114779	2.35052
2.63E+09	0.113921	2.37296
2.64E+09	0.113244	2.39522
2.65E+09	0.112706	2.41733
2.66E+09	0.112275	2.43928
2.67E+09	0.111929	2.46109
2.68E+09	0.111655	2.48277
2.69E+09	0.111439	2.50434
2.70E+09	0.111276	2.52581
2.71E+09	0.111158	2.54718
2.72E+09	0.111081	2.56847
2.73E+09	0.111043	2.58969
2.74E+09	0.11104	2.61084
2.75E+09	0.111071	2.63193

2.76E+09	0.111135	2.65298
2.77E+09	0.111231	2.67399
2.78E+09	0.111358	2.69497
2.79E+09	0.111516	2.71593
2.80E+09	0.111706	2.73687
2.81E+09	0.111928	2.7578
2.82E+09	0.112181	2.77874
2.83E+09	0.112468	2.79968
2.84E+09	0.112788	2.82064
2.85E+09	0.113144	2.84162
2.86E+09	0.113537	2.86264
2.87E+09	0.113969	2.88371
2.88E+09	0.114441	2.90482
2.89E+09	0.114957	2.926
2.90E+09	0.115519	2.94725
2.91E+09	0.116131	2.96859
2.92E+09	0.116796	2.99002
2.93E+09	0.117519	3.01155
2.94E+09	0.118305	3.03321
2.95E+09	0.11916	3.05501
2.96E+09	0.120091	3.07696
2.97E+09	0.121106	3.09907
2.98E+09	0.122215	3.12137
2.99E+09	0.12343	3.14388
3.00E+09	0.124762	3.16662
3.01E+09	0.12623	3.18961
3.02E+09	0.127851	3.21288
3.03E+09	0.129651	3.23647
3.04E+09	0.131658	3.2604
3.05E+09	0.133907	3.28471
3.06E+09	0.136444	3.30945
3.07E+09	0.139326	3.33466
3.08E+09	0.142625	3.3604
3.09E+09	0.146435	3.38672
3.10E+09	0.150878	3.41368
3.11E+09	0.156117	3.44133
3.12E+09	0.162367	3.46973
3.13E+09	0.169919	3.49891
3.14E+09	0.179166	3.52883
3.15E+09	0.190628	3.55934
3.16E+09	0.204978	3.59008
3.17E+09	0.223009	3.62027
3.18E+09	0.245464	3.64844
3.19E+09	0.272504	3.67213
3.20E+09	0.302603	3.68793
3.21E+09	0.33116	3.69293
3.22E+09	0.350797	3.68804

3.23E+09	0.355722	3.68026
3.24E+09	0.346961	3.6792
3.25E+09	0.331324	3.69043
3.26E+09	0.315767	3.7138
3.27E+09	0.304283	3.74642
3.28E+09	0.2983	3.78542
3.29E+09	0.298006	3.82886
3.30E+09	0.303277	3.87572
3.31E+09	0.314112	3.92557
3.32E+09	0.330822	3.97836
3.33E+09	0.354133	4.03424
3.34E+09	0.385283	4.09338
3.35E+09	0.426163	4.15589
3.36E+09	0.479518	4.22152
3.37E+09	0.549216	4.28929
3.38E+09	0.640342	4.35672
3.39E+09	0.759983	4.41856
3.40E+09	0.915762	4.46413
3.41E+09	1.11311	4.47402
3.42E+09	1.34707	4.41751
3.43E+09	1.58743	4.25833
3.44E+09	1.76797	3.98111
3.45E+09	1.81196	3.63025
3.46E+09	1.69722	3.30356
3.47E+09	1.47942	3.0797
3.48E+09	1.23667	2.97123
3.49E+09	1.01811	2.94833
3.50E+09	0.839926	2.97584
3.51E+09	0.700795	3.02857
3.52E+09	0.593688	3.09173
3.53E+09	0.511205	3.15751
3.54E+09	0.447211	3.22209
3.55E+09	0.397034	3.2838
3.56E+09	0.357232	3.34202
3.57E+09	0.32529	3.39671
3.58E+09	0.299371	3.44805
3.59E+09	0.278121	3.49632
3.60E+09	0.260531	3.54186
3.61E+09	0.245846	3.58495
3.62E+09	0.233491	3.62589
3.63E+09	0.223022	3.66493
3.64E+09	0.214097	3.70229
3.65E+09	0.206445	3.73818
3.66E+09	0.199855	3.77276
3.67E+09	0.194154	3.80619
3.68E+09	0.189206	3.83859
3.69E+09	0.184899	3.87009

3.70E+09	0.18114	3.90079
3.71E+09	0.177854	3.93076
3.72E+09	0.17498	3.9601
3.73E+09	0.172464	3.98886
3.74E+09	0.170263	4.01712
3.75E+09	0.168339	4.04492
3.76E+09	0.166661	4.07232
3.77E+09	0.165203	4.09935
3.78E+09	0.163941	4.12606
3.79E+09	0.162856	4.15248
3.80E+09	0.161931	4.17865
3.81E+09	0.161151	4.20458
3.82E+09	0.160504	4.23032
3.83E+09	0.159978	4.25588
3.84E+09	0.159564	4.28129
3.85E+09	0.159253	4.30657
3.86E+09	0.159039	4.33173
3.87E+09	0.158914	4.3568
3.88E+09	0.158874	4.38179
3.89E+09	0.158913	4.40672
3.90E+09	0.159028	4.43159
3.91E+09	0.159214	4.45643
3.92E+09	0.159469	4.48125
3.93E+09	0.15979	4.50606
3.94E+09	0.160175	4.53088
3.95E+09	0.160622	4.55571
3.96E+09	0.161131	4.58056
3.97E+09	0.161699	4.60546
3.98E+09	0.162326	4.6304
3.99E+09	0.163013	4.6554
4.00E+09	0.163759	4.68047
4.01E+09	0.164566	4.70562
4.02E+09	0.165435	4.73086
4.03E+09	0.166367	4.7562
4.04E+09	0.167365	4.78166
4.05E+09	0.168433	4.80724
4.06E+09	0.169575	4.83295
4.07E+09	0.170796	4.85881
4.08E+09	0.172104	4.88484
4.09E+09	0.173508	4.91103
4.10E+09	0.175019	4.93742
4.11E+09	0.176653	4.964
4.12E+09	0.17843	4.9908
4.13E+09	0.180374	5.01783
4.14E+09	0.18252	5.0451
4.15E+09	0.184912	5.07263
4.16E+09	0.187608	5.10041

4.17E+09	0.190686	5.12843
4.18E+09	0.194244	5.15666
4.19E+09	0.198406	5.18502
4.20E+09	0.203308	5.21334
4.21E+09	0.209068	5.24136
4.22E+09	0.215698	5.26863
4.23E+09	0.222941	5.29457
4.24E+09	0.230074	5.31864
4.25E+09	0.235897	5.34081
4.26E+09	0.239237	5.36194
4.27E+09	0.239748	5.3836
4.28E+09	0.238166	5.40713
4.29E+09	0.235694	5.43301
4.30E+09	0.233303	5.461
4.31E+09	0.231501	5.49061
4.32E+09	0.23045	5.52137
4.33E+09	0.230133	5.55295
4.34E+09	0.230462	5.58513
4.35E+09	0.231343	5.6178
4.36E+09	0.232687	5.65089
4.37E+09	0.234426	5.68438
4.38E+09	0.236505	5.71826
4.39E+09	0.238884	5.75255
4.40E+09	0.241534	5.78724
4.41E+09	0.244433	5.82237
4.42E+09	0.247567	5.85795
4.43E+09	0.250926	5.89401
4.44E+09	0.254507	5.93057
4.45E+09	0.258307	5.96766
4.46E+09	0.262327	6.0053
4.47E+09	0.266571	6.04354
4.48E+09	0.271044	6.08238
4.49E+09	0.275755	6.12187
4.50E+09	0.280711	6.16204
4.51E+09	0.285925	6.20293
4.52E+09	0.291409	6.24456
4.53E+09	0.297176	6.28697
4.54E+09	0.303244	6.33021
4.55E+09	0.309629	6.37431
4.56E+09	0.316352	6.41932
4.57E+09	0.323434	6.46528
4.58E+09	0.330899	6.51225
4.59E+09	0.338774	6.56028
4.60E+09	0.347088	6.60941
4.61E+09	0.355872	6.65972
4.62E+09	0.365163	6.71127
4.63E+09	0.374999	6.76412

4.64E+09	0.385422	6.81834
4.65E+09	0.396481	6.87402
4.66E+09	0.408228	6.93124
4.67E+09	0.420721	6.99008
4.68E+09	0.434025	7.05065
4.69E+09	0.448211	7.11305
4.70E+09	0.463361	7.17739
4.71E+09	0.479564	7.24379
4.72E+09	0.49692	7.31239
4.73E+09	0.515542	7.38333
4.74E+09	0.535558	7.45677
4.75E+09	0.557112	7.53287
4.76E+09	0.580364	7.61182
4.77E+09	0.605502	7.69381
4.78E+09	0.632733	7.77907
4.79E+09	0.6623	7.86783
4.80E+09	0.694476	7.96035
4.81E+09	0.72958	8.05692
4.82E+09	0.767976	8.15784
4.83E+09	0.810089	8.26345
4.84E+09	0.856412	8.37411
4.85E+09	0.907521	8.49024
4.86E+09	0.964092	8.61226
4.87E+09	1.02692	8.74065
4.88E+09	1.09695	8.87591
4.89E+09	1.1753	9.01857
4.90E+09	1.2633	9.16921
4.91E+09	1.36257	9.32841
4.92E+09	1.47503	9.49676
4.93E+09	1.60303	9.6748
4.94E+09	1.74942	9.86302
4.95E+09	1.91766	10.0617
4.96E+09	2.11201	10.271
4.97E+09	2.33767	10.4904
4.98E+09	2.60102	10.7188
4.99E+09	2.90982	10.9539
5.00E+09	3.27346	11.1915
5.02E+09	3.94656	11.5368
5.03E+09	4.81235	11.8288
5.05E+09	5.91488	11.9875
5.06E+09	7.27348	11.8759
5.08E+09	8.825	11.2944
5.09E+09	10.3364	10.0392
5.11E+09	11.377	8.0804
5.12E+09	11.5215	5.75752
5.14E+09	10.7117	3.6652
5.15E+09	9.3137	2.23056

5.17E+09	7.78355	1.50134
5.18E+09	6.40384	1.29775
5.20E+09	5.27122	1.41281
5.21E+09	4.38134	1.69556
5.23E+09	3.69477	2.05478
5.24E+09	3.16824	2.44078
5.26E+09	2.76519	2.82845
5.27E+09	2.45774	3.20628
5.29E+09	2.22594	3.56972
5.30E+09	2.05649	3.91714
5.32E+09	1.94144	4.24636
5.33E+09	1.87498	4.55021
5.35E+09	1.84209	4.81441
5.36E+09	1.80475	5.03972
5.38E+09	1.74205	5.27173
5.39E+09	1.68736	5.54164
5.41E+09	1.6674	5.83749
5.42E+09	1.68492	6.1455
5.44E+09	1.73737	6.46158
5.45E+09	1.82437	6.78659
5.47E+09	1.94868	7.12305
5.48E+09	2.11614	7.47376
5.50E+09	2.33597	7.84112
5.51E+09	2.6215	8.22627
5.53E+09	2.99142	8.62782
5.54E+09	3.47135	9.0393
5.56E+09	4.09553	9.44449
5.57E+09	4.90732	9.80902
5.59E+09	5.95501	10.0662
5.60E+09	7.27459	10.0971
5.62E+09	8.84425	9.71402
5.63E+09	10.4997	8.68588
5.65E+09	11.858	6.87437
5.66E+09	12.4154	4.46895
5.68E+09	11.9105	2.04036
5.69E+09	10.5774	0.17364
5.71E+09	8.91557	-0.91648
5.72E+09	7.31965	-1.35438
5.74E+09	5.9653	-1.36945
5.75E+09	4.8806	-1.15268
5.77E+09	4.03182	-0.82676
5.78E+09	3.37102	-0.46112
5.80E+09	2.85439	-0.09208
5.81E+09	2.44694	0.262743
5.83E+09	2.12213	0.59579
5.84E+09	1.86029	0.904697
5.86E+09	1.64684	1.18969

5.87E+09	1.471	1.45219
5.89E+09	1.32468	1.69411
5.90E+09	1.20182	1.91747
5.92E+09	1.09778	2.12422
5.93E+09	1.00899	2.31617
5.95E+09	0.932692	2.49494
5.96E+09	0.866701	2.66196
5.98E+09	0.8093	2.81853
5.99E+09	0.759115	2.96576
6.01E+09	0.71504	3.10465
6.02E+09	0.676178	3.23605
6.04E+09	0.641797	3.36075
6.05E+09	0.611297	3.47941
6.07E+09	0.584186	3.59264
6.08E+09	0.560056	3.70097
6.10E+09	0.538569	3.80488
6.11E+09	0.51945	3.90481
6.13E+09	0.502471	4.00113
6.14E+09	0.48745	4.0942
6.16E+09	0.474242	4.18435
6.17E+09	0.462738	4.27187
6.19E+09	0.452863	4.35703
6.20E+09	0.444577	4.44008
6.22E+09	0.437874	4.52126
6.23E+09	0.432785	4.60079
6.25E+09	0.429387	4.67887
6.26E+09	0.427808	4.75568
6.28E+09	0.42824	4.83138
6.29E+09	0.430951	4.90607
6.31E+09	0.436308	4.97983
6.32E+09	0.444796	5.0526
6.34E+09	0.457036	5.12416
6.35E+09	0.473799	5.19405
6.37E+09	0.495958	5.26135
6.38E+09	0.524357	5.32448
6.40E+09	0.559445	5.38094
6.41E+09	0.600564	5.42718
6.43E+09	0.644827	5.45907
6.44E+09	0.686098	5.47348
6.46E+09	0.715538	5.47127
6.47E+09	0.725174	5.45942
6.49E+09	0.712884	5.44921
6.50E+09	0.683881	5.45047
6.52E+09	0.646953	5.46725
6.53E+09	0.609662	5.49829
6.55E+09	0.576304	5.53994
6.56E+09	0.548419	5.58868

6.58E+09	0.526132	5.64205
6.59E+09	0.509215	5.6986
6.61E+09	0.497598	5.75748
6.62E+09	0.49145	5.818
6.64E+09	0.491098	5.87944
6.65E+09	0.496957	5.941
6.67E+09	0.509521	6.00171
6.68E+09	0.529346	6.06029
6.70E+09	0.556714	6.11456
6.71E+09	0.592244	6.16302
6.73E+09	0.63566	6.20204
6.74E+09	0.684852	6.22718
6.76E+09	0.734863	6.23426
6.77E+09	0.7776	6.22151
6.79E+09	0.803545	6.19212
6.80E+09	0.805696	6.15504
6.82E+09	0.783364	6.1221
6.83E+09	0.742445	6.103
6.85E+09	0.692026	6.10208
6.86E+09	0.640474	6.1186
6.88E+09	0.59346	6.14891
6.89E+09	0.553874	6.18855
6.91E+09	0.522522	6.23335
6.92E+09	0.498828	6.27992
6.94E+09	0.481337	6.32578
6.95E+09	0.468121	6.36945
6.97E+09	0.45719	6.41045
6.98E+09	0.446898	6.44916
7.00E+09	0.436219	6.48646
7.01E+09	0.424781	6.52333
7.03E+09	0.412699	6.56057
7.04E+09	0.40034	6.59865
7.06E+09	0.388119	6.63777
7.07E+09	0.376395	6.67793
7.09E+09	0.365423	6.71902
7.10E+09	0.355362	6.76087
7.12E+09	0.346293	6.80332
7.13E+09	0.338245	6.84622
7.15E+09	0.33121	6.88945
7.16E+09	0.325165	6.93293
7.18E+09	0.32008	6.97658
7.19E+09	0.315924	7.02035
7.21E+09	0.312673	7.06422
7.22E+09	0.310312	7.10818
7.24E+09	0.308842	7.15219
7.25E+09	0.308274	7.19628
7.27E+09	0.308639	7.24042

7.28E+09	0.309983	7.2846
7.30E+09	0.312363	7.3288
7.31E+09	0.315835	7.37295
7.33E+09	0.320429	7.41697
7.34E+09	0.326116	7.4608
7.36E+09	0.332804	7.50447
7.37E+09	0.340464	7.54824
7.39E+09	0.349392	7.59252
7.40E+09	0.360366	7.63759
7.42E+09	0.374451	7.6832
7.43E+09	0.392662	7.72855
7.45E+09	0.415822	7.77239
7.46E+09	0.444504	7.81299
7.48E+09	0.478792	7.84796
7.49E+09	0.517735	7.87426
7.51E+09	0.558555	7.88865
7.52E+09	0.596112	7.88907
7.54E+09	0.623651	7.87662
7.55E+09	0.635501	7.85674
7.57E+09	0.630275	7.83749
7.58E+09	0.611514	7.8257
7.60E+09	0.585008	7.82425
7.61E+09	0.555538	7.8325
7.63E+09	0.525644	7.84859
7.64E+09	0.496391	7.87107
7.66E+09	0.468539	7.89909
7.67E+09	0.442929	7.93184
7.69E+09	0.420225	7.96828
7.70E+09	0.400691	8.00726
7.72E+09	0.38422	8.04781
7.73E+09	0.370493	8.08916
7.75E+09	0.359115	8.13083
7.76E+09	0.349703	8.1725
7.78E+09	0.341919	8.214
7.79E+09	0.335482	8.25521
7.81E+09	0.330163	8.2961
7.82E+09	0.325781	8.33665
7.84E+09	0.322191	8.37684
7.85E+09	0.319279	8.4167
7.87E+09	0.316951	8.45621
7.88E+09	0.315126	8.49537
7.90E+09	0.313722	8.53416
7.91E+09	0.31264	8.57257
7.93E+09	0.311747	8.61056
7.94E+09	0.310859	8.64818
7.96E+09	0.309773	8.68559
7.97E+09	0.308383	8.72312

7.99E+09	0.306861	8.76116
8.00E+09	0.305686	8.79993
8.02E+09	0.305384	8.83923
8.03E+09	0.306237	8.87858
8.05E+09	0.308237	8.91747
8.06E+09	0.31118	8.95543
8.08E+09	0.314711	8.99209
8.09E+09	0.318298	9.02716
8.11E+09	0.321215	9.06057
8.12E+09	0.322652	9.09265
8.14E+09	0.322004	9.12419
8.15E+09	0.319186	9.15622
8.17E+09	0.314698	9.18966
8.18E+09	0.309357	9.22493
8.20E+09	0.303931	9.26199
8.21E+09	0.298938	9.30051
8.23E+09	0.294631	9.34012
8.24E+09	0.291077	9.38049
8.26E+09	0.288241	9.42135
8.27E+09	0.286049	9.46256
8.29E+09	0.284419	9.50401
8.30E+09	0.283274	9.54563
8.32E+09	0.28255	9.58739
8.33E+09	0.282198	9.62929
8.35E+09	0.282177	9.6713
8.36E+09	0.282459	9.71344
8.38E+09	0.282937	9.75545
8.39E+09	0.28365	9.7977
8.41E+09	0.284611	9.84018
8.42E+09	0.285835	9.88291
8.44E+09	0.287338	9.92589
8.45E+09	0.289137	9.96911
8.47E+09	0.291253	10.0126
8.48E+09	0.293708	10.0563
8.50E+09	0.296529	10.1003
8.51E+09	0.299747	10.1445
8.53E+09	0.303402	10.1889
8.54E+09	0.307538	10.2336
8.56E+09	0.312216	10.2786
8.57E+09	0.317506	10.3237
8.59E+09	0.323502	10.3691
8.60E+09	0.330313	10.4145
8.62E+09	0.338077	10.46
8.63E+09	0.346946	10.5054
8.65E+09	0.357082	10.5505
8.66E+09	0.368623	10.5949
8.68E+09	0.381631	10.6381

8.69E+09	0.395989	10.6795
8.71E+09	0.411279	10.7183
8.72E+09	0.426645	10.7538
8.74E+09	0.440756	10.7858
8.75E+09	0.451997	10.8144
8.77E+09	0.458929	10.841
8.78E+09	0.460858	10.8673
8.80E+09	0.458117	10.8955
8.81E+09	0.451884	10.9269
8.83E+09	0.443666	10.9622
8.84E+09	0.434831	11.0013
8.86E+09	0.426369	11.0437
8.87E+09	0.418873	11.0889
8.89E+09	0.412634	11.1363
8.90E+09	0.407742	11.1853
8.92E+09	0.404183	11.2356
8.93E+09	0.401895	11.287
8.95E+09	0.4008	11.3392
8.96E+09	0.40083	11.3921
8.98E+09	0.401931	11.4456
8.99E+09	0.404069	11.4997
9.01E+09	0.407232	11.5542
9.02E+09	0.411422	11.6091
9.04E+09	0.41666	11.6644
9.05E+09	0.422964	11.7199
9.07E+09	0.430342	11.7754
9.08E+09	0.438759	11.8308
9.10E+09	0.448105	11.8859
9.11E+09	0.458146	11.9404
9.13E+09	0.4685	11.9941
9.14E+09	0.47865	12.047
9.16E+09	0.488047	12.0995
9.17E+09	0.496279	12.1521
9.19E+09	0.503246	12.2056
9.20E+09	0.509218	12.2606
9.22E+09	0.514749	12.3178
9.23E+09	0.520495	12.3773
9.25E+09	0.527066	12.4392
9.26E+09	0.534945	12.5032
9.28E+09	0.54449	12.5692
9.29E+09	0.555959	12.6368
9.31E+09	0.569558	12.7059
9.32E+09	0.585461	12.7762
9.34E+09	0.603837	12.8476
9.35E+09	0.62485	12.9199
9.37E+09	0.648662	12.9927
9.38E+09	0.675431	13.066

9.40E+09	0.705313	13.1393
9.41E+09	0.738474	13.2125
9.43E+09	0.775102	13.2852
9.44E+09	0.815415	13.357
9.46E+09	0.859652	13.4273
9.47E+09	0.908022	13.4955
9.49E+09	0.960612	13.5605
9.50E+09	1.01724	13.621
9.52E+09	1.07726	13.6756
9.53E+09	1.13936	13.7227
9.55E+09	1.20137	13.761
9.56E+09	1.26028	13.7898
9.58E+09	1.3125	13.8098
9.59E+09	1.3545	13.8233
9.61E+09	1.38373	13.8343
9.62E+09	1.39948	13.8477
9.64E+09	1.40321	13.8684
9.65E+09	1.39824	13.9001
9.67E+09	1.38885	13.945
9.68E+09	1.37931	14.003
9.70E+09	1.37307	14.0729
9.71E+09	1.37243	14.1526
9.73E+09	1.37849	14.2399
9.74E+09	1.39136	14.333
9.76E+09	1.41054	14.4308
9.77E+09	1.43541	14.533
9.79E+09	1.46574	14.6402
9.80E+09	1.50198	14.7533
9.82E+09	1.5453	14.8733
9.83E+09	1.59746	15.0006
9.85E+09	1.66052	15.1353
9.86E+09	1.73673	15.2773
9.88E+09	1.82849	15.4258
9.89E+09	1.93834	15.5799
9.91E+09	2.06913	15.7385
9.92E+09	2.22409	15.8999
9.94E+09	2.40693	16.0616
9.95E+09	2.62191	16.2205
9.97E+09	2.87374	16.3716
9.98E+09	3.16743	16.5083
1.00E+10	3.50775	16.621
1.00E+10	3.63224	16.6511
